# Supplementary figures and images for: Validation of a commercially available mobile application for velocity-based resistance training
Source: PeerJ. 2024 Jul 24;12:e17789. doi: 10.7717/peerj.17789 (PMC11283170; doi:10.7717/peerj.17789)

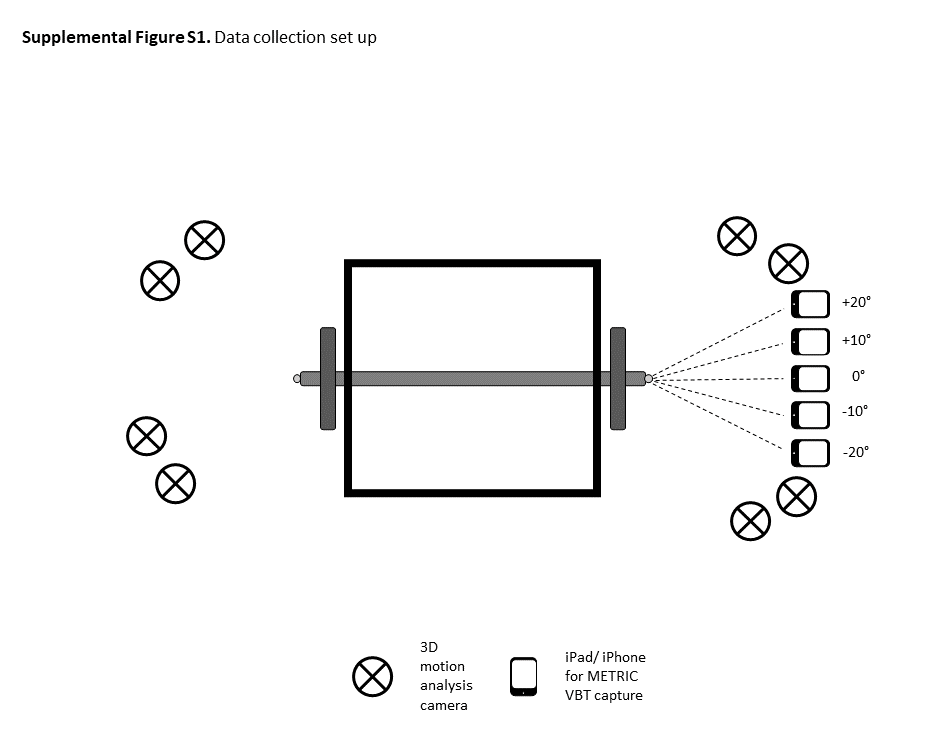

Supplement: Supplemental Information 1 — Diagram credit: Claire Kenneally-Dabrowski [file peerj-12-17789-s001.png]
