# Supplementary material for: Validation of a commercially available mobile application for velocity-based resistance training
Source: PeerJ. 2024 Jul 24;12:e17789. doi: 10.7717/peerj.17789 (PMC11283170; doi:10.7717/peerj.17789)
Supplement: Supplemental Information 2 [file peerj-12-17789-s002.docx]

**Supplemental Data S2: Extended Bland-Altman results**

**d)**

**Squat: RoM**

**b)**

**a)**

|  |  |
| --- | --- |
| **c)** | **d)** |
| 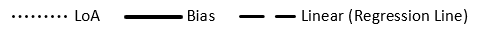 | |
| **Figure 1.** Bland-Altman analyses displayed the mean bias and limits of agreement (LoA) between the Metric VBT application at the a) -20° b) -10° c) +20° and d) +10° camera positions and Vicon when measuring repetition RoM during the back squat. | |

**Squat: Velocity**

**b)**

| **b)**  **a)** |  |
| --- | --- |
| **d)**  **c)** |  |
| 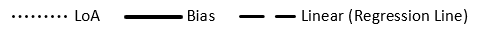 | |
| **Figure 2.** Bland-Altman analyses displayed the mean bias and limits of agreement (LoA) between the Metric VBT application at the a) -20° b) -10° c) +20° and d) +10° camera positions and Vicon when measuring average repetition velocity during the back squat. | |

**Bench Press: RoM**

**c)**

**b)**

**a)**

|  |  |
| --- | --- |
| **d)** |  |
| 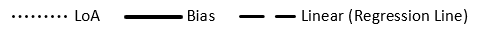 | |
| **Figure 3.** Bland-Altman analyses displayed the mean bias and limits of agreement (LoA) between the Metric VBT application at the a) -20° b) -10° c) +20° and d) +10° camera positions and Vicon when measuring repetition RoM during the bench press. | |

**Bench Press: Velocity**

**b)**

**c)**

**a)**

|  |  |
| --- | --- |
|  | **d)** |
| 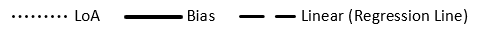 | |
| **Figure 4.** Bland-Altman analyses displayed the mean bias and limits of agreement (LoA) between the Metric VBT application at the a) -20° b) -10° c) +20° and d) +10° camera positions and Vicon when measuring average repetition velocity during the bench press. | |
